# Metabolic Effects of the Waist-To-Hip Ratio Associated Locus *GRB14/COBLL1* Are Related to *GRB14* Expression in Adipose Tissue

**DOI:** 10.3390/ijms23158558

**Published:** 2022-08-02

**Authors:** Chang Sun, Franz Förster, Beate Gutsmann, Yusef Moulla, Christine Stroh, Arne Dietrich, Michael R. Schön, Daniel Gärtner, Tobias Lohmann, Miriam Dressler, Michael Stumvoll, Matthias Blüher, Peter Kovacs, Jana Breitfeld, Esther Guiu-Jurado

**Affiliations:** 1 Medical Department III—Endocrinology, Nephrology, Rheumatology, University of Leipzig Medical Center, 04103 Leipzig, Germany; sunchang141@gmail.com (C.S.); franzfoerster@gmx.de (F.F.); beate.gutsmann@medizin.uni-leipzig.de (B.G.); michael.stumvoll@uniklinik-leipzig.de (M.S.); bluma@medizin.uni-leipzig.de (M.B.); peter.kovacs@medizin.uni-leipzig.de (P.K.); jana.breitfeld@medizin.uni-leipzig.de (J.B.); 2 Clinic for Visceral, Transplantation and Thorax and Vascular Surgery, University Hospital Leipzig, 04103 Leipzig, Germany; yusef.moulla@medizin.uni-leipzig.de (Y.M.); arne.dietrich@medizin.uni-leipzig.de (A.D.); 3 Departement of Obesity and Metabolic Surgery, SRH Wald-Klinikum Gera Str.d. Friedens 122, 07548 Gera, Germany; christine.stroh@srh.de; 4 Städtisches Klinikum Karlsruhe, Clinic of Visceral Surgery, 76133 Karlsruhe, Germany; m.schoen@klinikum-karlsruhe.de (M.R.S.); daniel.gaertner@klinikum-karlsruhe.de (D.G.); 5 Municipal Clinic Dresden-Neustadt, 01129 Dresden, Germany; tobias.lohmann@klinikum-dresden.de (T.L.); miriam.dressler@klinikum-dresden.de (M.D.); 6 Helmholtz Institute for Metabolic, Obesity and Vascular Research (HI-MAG) of the Helmholtz Zentrum München at the University of Leipzig and University Hospital Leipzig, 04103 Leipzig, Germany; 7 Deutsches Zentrum für Diabetesforschung e.V., 85764 Neuherberg, Germany

**Keywords:** *COBLL1*, *GRB14*, rs10195252, rs6738627, adipose tissue

## Abstract

*GRB14/COBLL1* locus has been shown to be associated with body fat distribution (FD), but neither the causal gene nor its role in metabolic diseases has been elucidated. We hypothesize that *GRB14/COBLL1* may act as the causal genes for FD-related SNPs (rs10195252 and rs6738627), and that they may be regulated by SNP to effect obesity-related metabolic traits. We genotyped rs10195252 and rs6738627 in 2860 subjects with metabolic phenotypes. In a subgroup of 560 subjects, we analyzed *GRB14/COBLL1* gene expression in paired visceral and subcutaneous adipose tissue (AT) samples. Mediation analyses were used to determine the causal relationship between SNPs, AT *GRB14/COBLL1* mRNA expression, and obesity-related traits. In vitro gene knockdown of *Grb14/Cobll1* was used to test their role in adipogenesis. Both gene expressions in AT are correlated with waist circumference. Visceral *GRB14* mRNA expression is associated with FPG and HbA1c. Both SNPs are associated with triglycerides, FPG, and leptin levels. Rs10195252 is associated with HbA1c and seems to be mediated by visceral AT *GRB14* mRNA expression. Our data support the role of the *GRB14/COBLL1* gene expression in body FD and its locus in metabolic sequelae: in particular, lipid metabolism and glucose homeostasis, which is likely mediated by AT *GRB14* transcript levels.

## 1. Introduction

The prevalence of obesity worldwide has increased dramatically, and intervention efforts for weight loss, including calorie restriction and increased physical activity, have only been able to counteract this trend to a small extent [1]. Despite recent advances, the underlying mechanisms making some individuals more susceptible to obesity and related comorbidities remain unknown. Many studies clearly support the role of genetics in obesity and adverse body FD, which has been acknowledged as a major predictor of obesity-associated metabolic sequelae [2,3,4,5].

Heid et al. performed meta-GWAS for waist–hip ratio (WHR) in 2010, which revealed the single nucleotide polymorphism (SNP) rs10195252 located near the Growth Factor Receptor-Bound Protein 14 (*GRB14*) gene with evident sexual dimorphism [6]. In this case, the association with WHR was more pronounced in women, and the T-allele carriers had increased *GRB14* expression in subcutaneous (sc) and omental adipose tissue (AT). In addition, an association with triglycerides (TG), insulin levels, and high-density (HDL) cholesterol was reported [6,7]. Shungin et al. proposed *GRB14*, but also Cordon-Bleu WH2 Repeat Protein Like 1 (*COBLL1*) as potential candidate gene affected by the WHR-associated SNPs in 2015 [8]. Similar to *GRB14*, the SNP mapping within the *COBLL1* gene showed marked sexual dimorphism, with stronger effects on WHR in women. Moreover, another GWAS meta-analysis reported that rs6738627 located in *COBLL1* and nearby *GRB14* had larger effects on body fat percentage than on BMI, suggesting a specific effect on adiposity rather than total body mass [9]. Interestingly, rs6738627 is in high LD (r^2^ = 0.81) with the WHR-associated rs10195252. Thus, based on the concomitant and directionally consistent associations with insulin-resistance-related traits and plausible mechanisms of action, the *COBLL1/GRB14* locus has become particularly attractive for follow up on potential target genes explaining the association of SNPs within this locus with WHR [10].

GRB14 inhibits insulin receptor (IR) signaling [11] and, consequently, the phosphorylation of downstream signaling peptides like protein kinase B (PKB, also called AKT) or extracellular signal-regulated kinase (ERK) [12,13]. Furthermore, a drastic decrease in insulin induced processing and expression of sterol regulatory element-binding protein 1c (*SREBP-1c*) was reported as a consequence of *Grb14* knock-down in murine primary hepatocytes in 2008 [14]. COBLL1 has not been extensively investigated so far. It has been proposed to be involved in the development of neural tubes [15] and has been reported to be associated with several types of cancers [16,17]. Chen et al. showed that *GRB14* knock-out resulted in decreased differentiation efficiency and proliferation rate, along with reduced lipid storage, and *COBLL1* knock-out caused excessive lipid storage and lipolysis but did not affect adipogenesis or insulin-stimulated AKT2 phosphorylation in human preadipocytes [18].

Given the robustly replicated SNP associations with WHR, waist and hip circumference and body fat percentage, as well as the known physiological function of *GRB14* and *COBLL1*, we measured the expression of these genes in human visceral (vis) and sc AT and analyzed their correlations with obesity-related metabolic traits. Furthermore, we investigated their functional relevance in adipogenesis in vitro and tested the causal relationship between genetic variants and metabolic traits.

## 2. Results

### 2.1. GRB14 and COBLL1 mRNA Expression in AT Correlates with Parameters of Obesity and Body Fat Distribution

**Figure 1:**
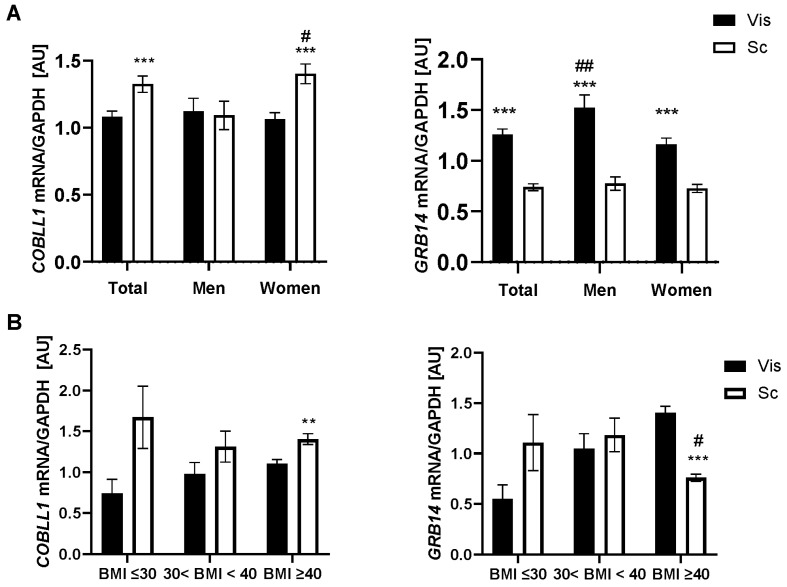
(**A**) *COBLL1* and *GRB14* mRNA expression in 524/560 paired human visceral (vis) and subcutaneous (sc) adipose tissue (AT) samples in the total cohort and grouped by sex (131/149 men, 393/411 women). Data are shown as mean ± SEM; *** for comparisons between sc and vis fat depot expression; # for comparison between men and women in the same AT depot; # *p* < 0.05; ## *p*< 0.01; *** *p* < 0.001. (**B**) *COBLL1* and *GRB14* mRNA expression in paired human visceral (vis) and subcutaneous (sc) adipose tissue (AT) samples grouped by obesity status (BMI < 30 kg/m^2^, *n* = 19; 30 ≤ BMI < 40 kg/m^2^, *n* = 47; BMI ≥ 40 kg/m^2^, *n* = 454). Mean ± SEM; ** sc vs. vis AT depot; # 30–40 kg/m^2^ ≥ 40 kg/m^2^ vs. <30 kg/m^2^ depot; # *p* < 0.05; ** *p* < 0.01; *** *p* < 0.001.

Given the known sexual dimorphism of genetic variants associated with WHR and waist circumference [6,8], we analyzed mRNA expression data in the entire cohort and separately for men and women. Overall, *COBLL1* mRNA expression was significantly higher in sc than in vis, specifically for women (*p* < 0.001), while *GRB14* mRNA expression was higher in vis than sc and higher for men than women (*p* < 0.001; Figure 1A). Therefore, sex was controlled for in all subsequent analyses; all *p*-values were adjusted for age, sex, and BMI.

Changes in gene/pathway expression activity in AT may be related to a certain FD pattern or to metabolic traits as a cause or consequence. Therefore, we compared mRNA expression between BMI < 30 kg/m^2^, BMI: 30–40 kg/m^2^, and BMI ≥ 40 kg/m^2^ subjects. Except for *GRB14*, whose mRNA expression was lower in sc AT of individuals with BMI ≥ 40 kg/m^2^ compared to those with BMI < 30 kg/m^2^ (*p* < 0.05), none of the genes showed significant differences in AT mRNA levels between subjects with BMI < 30 kg/m^2^ and BMI: 30–40 kg/m^2^ (<30 kg/m^2^ vs. 30–40 kg/m^2^ vs. ≥40 kg/m^2^: 19 vs. 47 vs. 454) (Figure 1B). Although not significant, vis *COBLL1* and vis *GRB14* mRNA expression tended to be higher with increasing BMI. Surprisingly, only in patients with BMI ≥ 40 kg/m^2^, *GRB14* gene expression was lower (*p* < 0.001), and *COBLL1* mRNA expression was increased in sc AT compared to its expression in vis AT (*p* < 0.01; Figure 1B). For individual subjects, *GRB14* and *COBLL1* mRNA expression in vis versus sc AT depots was correlated even after adjustment (p_adj_) for BMI, sex, and age (r = 0.16, *p* < 0.001, p_adj_ < 0.001; r = 0.13, *p* < 0.01, p_adj_ < 0.001).

**Figure 2:**
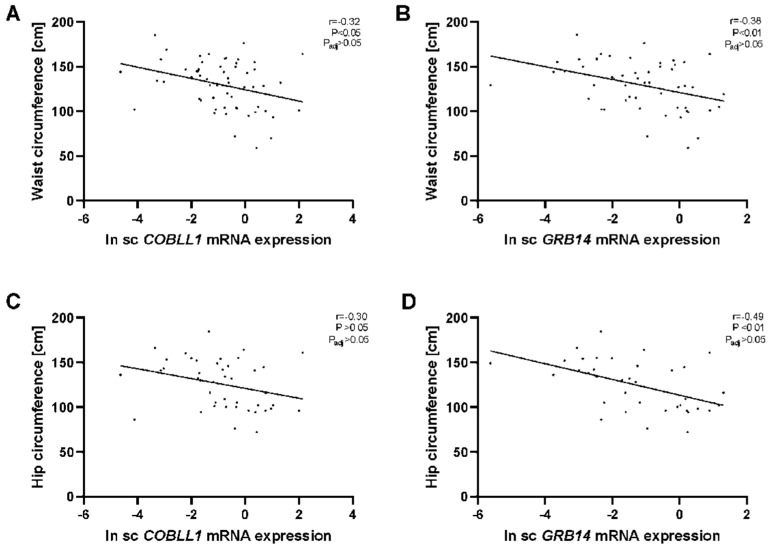
Correlation between AT *COBLL1/GRB14* mRNA expression and fat distribution variables. The correlation of natural log transformed sc *COBLL1/GRB14* mRNA levels with waist circumference (**A**,**B**, *n* = 59/53) and hip circumference (**C**,**D**, *n* = 43/37) is shown. Pearson correlation coefficients and corresponding *p*-value have been included; p_adj_: adjusted by BMI, age, and sex; sc: subcutaneous adipose tissue.

Moreover, we found a significant correlation between sc *COBLL1* and *GRB14* mRNA expression with waist circumference (Figure 2A,B) and that sc *GRB14* mRNA correlated with hip circumference but did not correlate with sc *COBLL1* expression (Figure 2C,D). However, these correlations did not withstand adjustment for BMI. On the other hand, vis *COBLL1/GRB14* mRNA expression did not correlate significantly with body FD parameters.

### 2.2. GRB14 and COBLL1 mRNA Expression in AT Correlates with Diabetes and Parameters of Glucose

**Figure 3:**
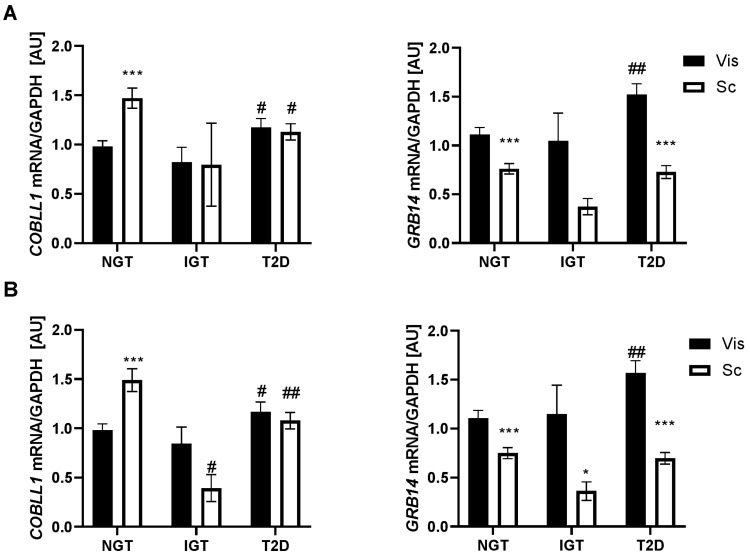
(**A**) *COBLL1* and *GRB14* mRNA expression in paired human visceral (vis) and subcutaneous (sc) adipose tissue (AT) samples grouped by type 2 diabetes status (NGT: subjects with normal glucose tolerance, *n* = 238; IGT: subjects with impaired glucose tolerance, *n* = 10; subjects with Type 2 diabetes, *n* = 197). Mean ± SEM; *vis vs. sc AT depot; #IGT/T2D vs. NGT; # *p* < 0.05; ## *p* < 0.01; *** *p* < 0.001. (**B**) *COBLL1* and *GRB14* mRNA in paired human visceral (vis) and subcutaneous (sc) adipose tissue (AT) samples grouped by type 2 diabetes status in BMI ≥ 40 kg/m^2^ patients. (NGT: subjects with normal glucose tolerance, *n* = 200; IGT: subjects with impaired glucose tolerance, *n* = 9; T2D: subjects with diabetes, *n* = 175). Mean ± SEM; *vis vs. sc AT depot; #IGT/T2D vs. NGT; */# *p* < 0.05; ## *p* < 0.01; *** *p* < 0.001.

As the state of glucose homeostasis may influence transcript expression and since certain genes may regulate vis fat mass accumulation and contribute to the development of T2D, we analyzed *COBLL1* and *GRB14* mRNA expression in subjects with normal glucose tolerance (NGT), impaired glucose tolerance (IGT), and T2D. Compared to subjects with NGT, subjects with T2D had significantly higher vis *GRB14* mRNA levels (*p* < 0.01; Figure 3A) even after adjusting for BMI, age, and sex (*p* < 0.05). In contrast, *COBLL1* mRNA expression was significantly lower in sc and higher in vis AT in subjects with T2D (*p* < 0.05; Figure 3A). However, this appears to be driven by age rather than obesity, as the association remained significant after adjusting for BMI and sex (*p* < 0.05), but not when including age in the statistical model.

Interestingly, in the BMI ≥40 kg/m^2^ group, vis *GRB14* mRNA expression was higher in patients with T2D compared to subjects with NGT (*p* < 0.01). Consistent with previous results, *GRB14* mRNA expression was lower in sc AT compared to vis AT in BMI ≥ 40 kg/m^2^ group, even under different pre- and diabetes states (*p* < 0.001). The sc *COBLL1* gene expression was lower in subjects with IGT and T2D than in subjects with NGT (*p* < 0.05). In contrast, *COBLL1* mRNA expression was higher in sc AT than in vis AT in subjects with NGT (*p* < 0.001; Figure 3B), which seems to drive the difference between both fat depots in the cohort with BMI > 40 kg/m^2^ (Figure 1B).

**Figure 4:**
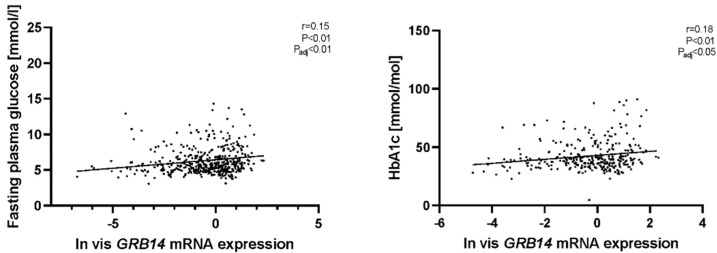
Correlation between vis AT *GRB14* mRNA expression glucose variables. The correlation of natural log transformed vis *GRB14* mRNA levels with fasting plasma glucose (*n* = 473) and glycated haemoglobin (HbA1c) (*n* = 309) is shown. Pearson correlation coefficients and corresponding *p*-value have been included; p_adj_: adjusted by BMI, age, and sex; vis: visceral adipose tissue.

We analyzed the association between *COBLL1* and *GRB14* mRNA expression and metabolic phenotypes, including diabetes, lipid, and inflammatory parameters, as well as adipokines (Table S1). We found that vis *GRB14* mRNA expression was correlated positively with glycated haemoglobin (HbA1c) and fasting plasma glucose (FPG) (Figure 4), even in multiple linear regression models after adjusting for age, sex, and BMI (p_adj_ < 0.05; Table S1). Sc *GRB14* mRNA expression was not significantly correlated with glucose variables. On the other hand, there was no significant association between *COBLL1* mRNA expression and the phenotypes studied in both AT (Table S1).

### 2.3. Both rs10195252 and rs6738627 Are Associated with Glucose and Lipid Metabolism, as well as Circulating Adipokines

To elucidate the possible association between the originally described WHR-associated genetic variant as well as the body fat-associated variant with human metabolic phenotypes [8,9], we genotyped the two SNPs rs10195252 T > C (*GRB14-COBLL1*) and rs6738627 G > A (*COBLL1*) in the entire cohort. Both SNPs were in Hardy–Weinberg Equilibrium (*p* > 0.05) with the following minor allele frequencies (MAFs): 43% for rs10195252 and 39% for rs6738627.

Carriers of the rs6738627 G-allele had a significantly higher risk of T2D compared with carriers of the A-allele (GG: OR 1.85; 95% CI [1.21–2.83], AG: OR 1.78; 95% CI [1.18–2.68], *p* < 0.01, adjusted for sex, age, and BMI; Table 1). Subsequently, given the association between the development of diabetes and increased BMI, we performed further analyses with stratifications for obesity status. The association between rs6738627 and risk of T2D remained significant even when stratified by BMI ≥ 40 kg/m^2^ (Table S2).

**Figure 5:**
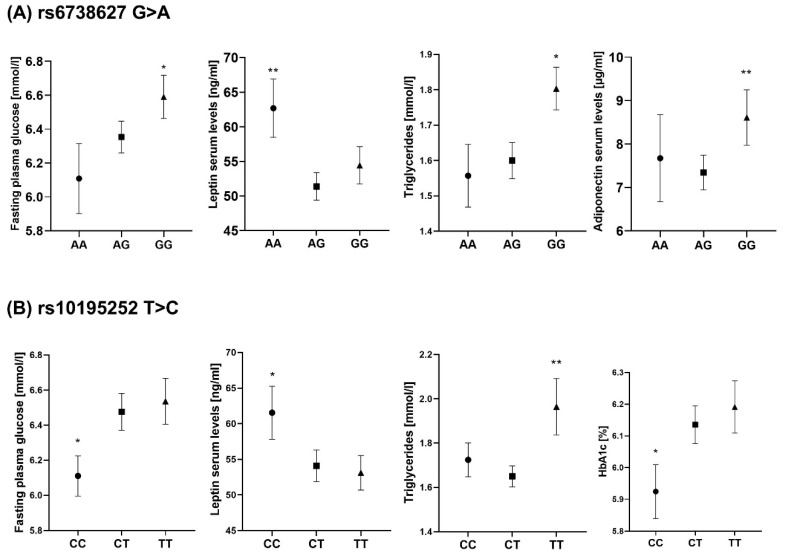
Association analyses of rs6738627 and rs10195252 with metabolic traits. (**A**) Association analyses of rs6738627 with FPG, leptin, TG, and adiponectin. (**B**) Association analyses of rs10195252 with FPG, leptin, TG, and HbA1c. * *p* < 0.05; ** *p* < 0.01; *p* adjusted for sex, age, and BMI.

Furthermore, rs6738627 and rs10195252 were significantly associated with FPG, as well as circulating TG and leptin (*p* < 0.05, adjusted for sex, age, BMI; Figure 5). Likewise, rs10195252 was significantly associated with HbA1c, and rs6738627 was associated with adiponectin levels (*p* < 0.05, adjusted for sex, age, BMI; Figure 5). Except for the above associations, no association was observed between the two SNPs and other phenotypes (Table S3).

### 2.4. Genotype-Specific mRNA Expression at Loci Associated with WHR and Body Fat

**Figure 6:**
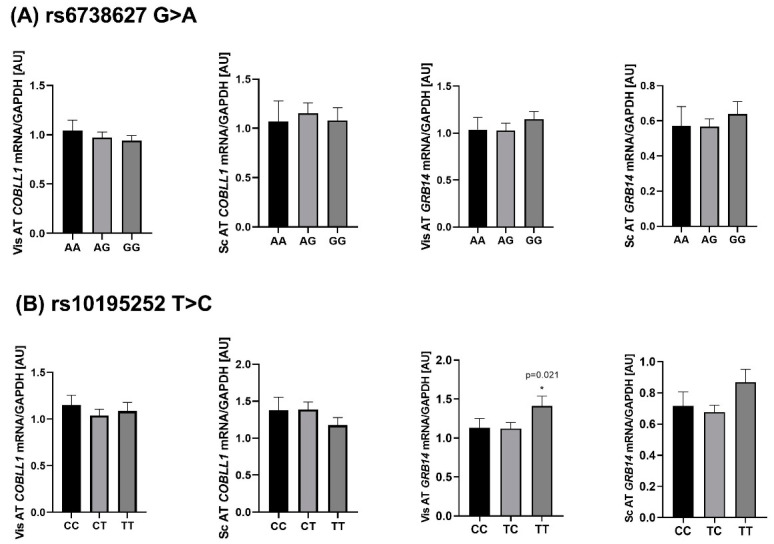
Association of rs6738627 and rs10195252 with *COBLL1* and *GRB14* mRNA expression in visceral (vis) and subcutaneous (sc) AT. (**A**) shows the rs6738627 associated with *COBLL1* and *GRB14* mRNA expression in both ATs. (**B**) shows the rs10195252 associated with *COBLL1* and *GRB14* mRNA expression in both ATs. Data are given as arithmetic mean ± SEM; *p* < 0.05 in additive mode of inheritance; adjusted for sex, age, BMI.

The eQTL (expression Quantitative Trait Loci) data can implicate regional transcripts that mediate trait associations and may help to sort out the expected direction. Therefore, we analyzed the relationship of rs10195252 and rs6738627 with AT gene expression. None of the SNPs showed association with *COBLL1* transcript quantity (Figure 6A). However, the rs10195252 T-allele was significantly associated with increased *GRB14* vis mRNA expression (*p* < 0.025 after adjusting for age, sex, and BMI; Figure 6B). Although not statistically significant, the rs10195252 T-allele carriers showed higher *GRB14* mRNA expression in sc AT, consistent with data previously reported by Schleinitz et al. [19].

### 2.5. Mediation Analyses

**Figure 7:**
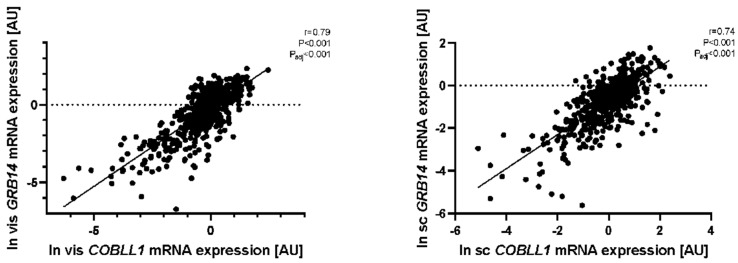
The correlation of *GRB14* and *COBLL1* mRNA expression in visceral (vis) and subcutaneous (sc) AT. The correlation of natural log transformed vis *COBLL1/GRB14* mRNA levels (*n* = 563) and sc *COBLL1/GRB14* mRNA (*n* = 486) are shown. Pearson correlation coefficients and corresponding *p*-value have been included; p_adj_: adjusted by BMI, age, and sex.

We applied mediation analyses between SNP, gene expression, and several obesity and metabolic traits. Mediation analyses revealed that the association between rs10195252 and HbA1c was fully mediated via vis *GRB14* gene expression (*p* < 0.05, Table S4). Although rs10195252 was associated with FPG, this appeared to be driven by other factors, as no significant association was found when *GRB14* mRNA expression was the mediating variable (*p* > 0.05, adjusted for sex, age, and BMI). Notably, *COBLL1* and *GRB14* mRNA expression were strongly correlated in both AT (Figure 7, Table S5). Although rs10195252 was not significantly associated with *COBLL1* mRNA expression in vis AT, mediation analyses revealed that rs10195252 had a significant direct effect on *COBLL1* expression (ß = −0.1496, *p* = 0.0057, adjusted for sex, age, and BMI). This may be due to the indirect effect from *GRB14* partially suppressing the direct effect of the SNP on *COBLL1* gene expression. On the other hand, the SNP also had a significant indirect effect on *GRB14* transcript levels, but not through *COBLL1* mRNA expression (*p* > 0.05, adjusted for sex, age, and BMI).

### 2.6. Decreased Lipid Accumulation in Grb14 Knockdown in Epididymal Cell Line

**Figure 8:**
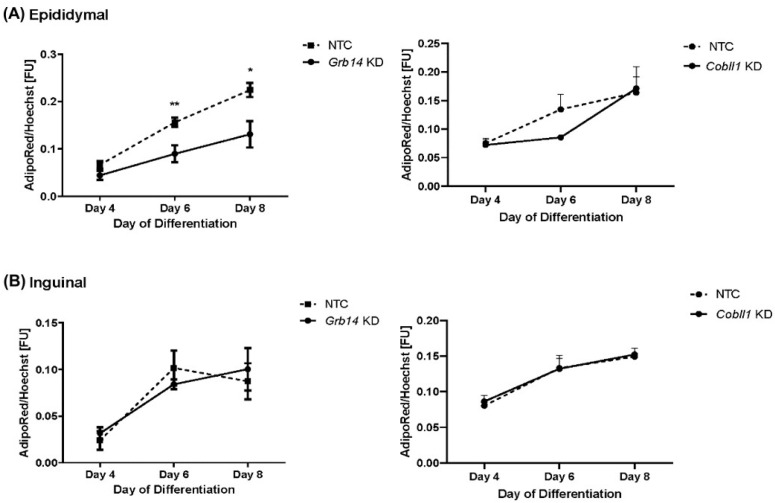
Quantification of lipid droplets by measuring the AdipoRedTM fluorescence signal normalized to the Hoechst fluorescence signal. Lipid droplets quantification (**A**) in the epididymal cell line and (**B**) in the inguinal cell line. Lipid accumulation normalized to the Hoechst signal compared to NTC (non-silencing siRNA group). AdipoRed stains the triglycerides of the lipid droplets and Hoechst stains the nuclei of cells. KD: knock-down. * *p* < 0.05, ** *p* < 0.01.

In vitro results showed that *Grb14*-knock-down (KD) significantly decreased lipid accumulation only in epididymal adipose tissue cells (*p* < 0.01 at Day 6 when tested vs. NTC controls) during adipogenesis, especially after 6 days of the induction of differentiation when the maximum lipid reduction was observed (Figure 8A). On the other hand, in inguinal adipose tissue cells, no significant change in lipid accumulation was observed when *Grb14* gene expression was decreased (Figure 8B). *Cobll1*-KD, in both adipose tissue (epididymal and inguinal) cell lines, did not affect lipid accumulation during adipogenesis (Figure 8).

## 3. Discussion

GWAS identified a large number of previously unrecognized genetic loci that may increase susceptibility to developing obesity, adverse fat distribution, and metabolic diseases. However, functional studies are warranted to clarify mechanisms that mediate the association between the genotype and phenotype. Our study focused on a WHR-associated locus harboring two candidate genes, *GRB14* and *COBLL1*, in the context of effects on obesity, fat distribution, and metabolic parameters. Our key findings are that *GRB14* and *COBLL1* mRNA expression is fat-depot-specific with differential expression between vis and sc AT and that these differences are gender sensitive. Furthermore, this correlates with measures of obesity/fat distribution and metabolic parameters related to T2D. Both vis and sc *GRB14/COBLL1* gene expression correlate with T2D, but vis *GRB14* expression is more of a risk factor for glucose metabolism because its mRNA expression is associated with HbA1c, FPG, and T2D independently of obesity. Moreover, rs10195252-T may present higher metabolic risk due to its association with increased transcript levels of the corresponding gene (*GRB14* mRNA levels in vis AT). Both SNPs (rs10195252 and rs6738627) were associated with triglycerides, FPG, and leptin serum levels. Rs6738627-G is associated with a higher risk of type 2 diabetes. Briefly, our data support the role of *GRB14/COBLL1* gene expression in body FD (vis/sc) and the role of the *GRB14/COBLL1* locus in metabolic sequelae of adverse body FD, particularly in lipid metabolism and glucose homeostasis and lipid metabolism, which is likely due to changes in *GRB14* expression in AT as suggested by the mediation analyses conducted here.

### 3.1. GRB14 and COBLL1 Expression Is Fat-Depot-Specific and Related to Obesity and T2D

It has been well-established that adverse body FD is associated with anthropometric and clinical outcomes such as metabolic and cardiovascular diseases [20,21,22]. Indeed, excess in vis adipose tissue imparts a greater risk of insulin resistance and T2D than excess of fat distributed subcutaneously [23,24]. In addition, sc AT seems to be protective against T2D, as its accumulation is associated with improved insulin sensitivity and beneficial lipid profile [25]. Here, we report significantly higher expression of *COBLL1* and *GRB14* in vis AT in patients with T2D, which seems to be independent of obesity. *GRB14* gene expression in vis AT was associated with FPG and HbA1c, further underscoring the potential role of GRB14 in the development of T2D. This may not appear surprising, considering that GRB14 is a multi-domain growth factor receptor bound protein that binds the insulin receptor as a pseudo substrate-inhibitor of the catalytic activity of the insulin receptor [11,12]. GRB14 regulates physiological activity of insulin as had been demonstrated in *Grb14* knockout mouse models [13,26,27], which showed improved glucose homeostasis and enhanced insulin signaling [26]. Consistent with these results, our in vitro results showed a decreased in lipid accumulation when we knock down *Grb14* in epididymal adipocytes. In humans, *GRB14* gene expression in skeletal muscle decreased after gastric bypass surgery accompanied by improved insulin sensitivity but increased in individuals with obesity [28]. Moreover, in vitro studies suggested the role of *GRB14* in adipogenesis as well [18]. Consistently, albeit not significantly, our present study showed positive correlation between the *GRB14* gene expression in vis AT and BMI.

In contrast to GRB14, the link between obesity related traits and COBLL1 is less clear. According to the gene ontology (GO) classification, *COBLL1* is recognized as actin-monomer-binding and cadherin-binding protein. Although GWAS have suggested COBLL1 as a potential determinant of obesity, T2D, and impaired lipid metabolism, functional data are rather scarce to date. However, in support of the COBLL1 role in metabolic disease, our study not only demonstrates fat-depot-specific *COBLL1* mRNA expression, but also a significantly lower *COBLL1* expression in sc AT of patients with T2D. Considering previously reported associations between *COBLL1* expression and lipid metabolism [18], our data suggest that the diminished *COBLL1* expression may be related to adipose tissue dysfunction.

It is noteworthy that sexual dimorphism has been reported in GWAS for FD. In line with this, *COBLL1* and *GRB14* expression in AT showed evident sexual dimorphisms as well. *COBLL1* expression, which was higher in sc AT compared to vis AT, was more pronounced in women. In contrast, *GRB14* expression was higher in vis AT compared to sc AT and characteristic for men. Sex hormones might influence *GRB14/COBLL1* expression in AT. Therefore, sex-specific effects need to be carefully considered in further studies aimed at clarifying the role of COBLL1 and GRB14 in adverse body FD and associated metabolic disorders.

### 3.2. Genetic Association of WHR-Related SNPs with Clinical Variables

It must be acknowledged that despite the clear evidence for strong correlations between *COBLL1* and *GRB14* mRNA expression levels in AT and metabolic traits related to obesity, the observed findings do not allow for drawing any conclusions regarding causal relationships between these entities. Therefore, mediation analyses such as, e.g., Mendelian randomization present a powerful tool to test the causal effect of a modifiable exposure on traits or diseases by using sequence variants in genes. Thus, we used this tool to test the associations of the reported WHR-SNPs on obesity and diabetes related metabolic traits. Rs10195252 and rs6738627 were as eQTLs for *GRB14* in esophagus-muscularis and for *COBLL1* in thyroid and brain-cerebellum tissues in GTEx database (www.gtexportal.org; accessed on 24 January 2022). Our data support rs10195252 as an eQTL for *GRB14* in visceral adipose tissue. The two SNPs in this study were significantly associated with TG, FPG and leptin serum levels, which in part confirmed previously reported data (particularly the association between rs10195252 and TG, and between rs6738627 and leptin levels) [29,30]. Of note, recent GWAS meta-analyses reported nominal evidence for sex heterogeneity for rs10195252 (p_heterogeneity_ = 0.039) with more effects on fasting insulin (FI) level in women than men [31]. In this study, we did not observe the association between rs10195252 and FI, even in the subgroup analyzed by gender. One possible reason is the limited statistical power to detect these associations (n_our_~1200 vs. n_GWAS_~96,000). Moreover, rs10195252 was associated with HbA1c and rs6738627 with adiponectin serum levels. Since both SNPs are in high LD (r^2^ = 0.81), the strong concordance of association results was not surprising. This is further emphasized by the relationship between the *COBLL1/GRB14* locus and FD and lipid metabolism. We therefore sought to clarify how the respective target genes of these variants contribute to the complex etiology of obesity and diabetes. Surprisingly, the rs6738627 showed significant association with T2D, as the risk of T2D for the homozygous G-allele carrier is 85% higher than for the homozygous A-allele carrier (Table 1). Furthermore, statistical analyses also presented an 83% increased risk of T2D for homozygous G-allele carriers in patients with BMI ≥ 40 kg/m^2^ (Table S2). Albeit not significantly associated with T2D in this study, rs10195252 was associated with T2D in previous GWAS, presenting a 7% higher risk only for T-allele carrier [32]. In summary, the SNP association analysis on rs6738627 and rs1019525 partly confirmed already known associations with traits within the loci reported by previous GWAS. Moreover, our study strengthened these findings by further genetic associations with numerous obesity-related metabolic traits (e.g., rs10195252 with FPG, leptin and HbA1c; rs6738627 with T2D, FPG, leptin, TG, and adiponectin). Altogether, both previous studies as well as the present study clearly point to the importance of the *GRB14/COBLL1* area in association with T2D and metabolism traits. Notably, rs6738627 has been reported as a potential candidate locus for T2D that deserves further attention [9].

Considering the relationship between *GRB14* and *COBLL1* mRNA expression in AT with obesity and diabetes-related parameters, as well as taking into account the above-mentioned SNP associations with metabolic traits, we conducted mediation analyses by statistically simulating the causal relationship between rs10195252 and *GRB14* mRNA expression in vis AT and HbA1c. We found *GRB14* gene expression in vis AT fully mediating the effects of rs10195252 on HbA1c (Table S4). Interestingly, there is a marked correlation between *COBLL1* and *GRB14* gene expression in AT. This may be due to the fact that *GRB14* and *COBLL1* are directly neighboring on the chromosome, and both genes might share the same promoters activating common transcriptional mechanisms (e.g., enhancer). In line with this, data from the public available database for the retrieval of interacting genes (STRING) implicated that the co-expression and interaction of *GRB14* and *COBLL1* may occur via gene fusions [33]. Our simulated data showed that *GRB14* gene expression in AT suppressed, at least in part, the direct effect of the rs10195252 on *COBLL1* gene expression. Considering the strong correlation of *GRB14/COBLL1* mRNA expression in AT and its potential function on lipid metabolism, we may need to consider the interaction of *COBLL1* and *GRB14* in adverse body FD and associated metabolic disorders in further studies.

Our study has some limitations that need to be acknowledged. Although we measured *COBLL1* and *GRB14* mRNA expression in a relatively high number (*n* = 560) of paired human sc and vis AT samples, we are aware that this sample size still limits the power of our study to detect weaker correlations. Similarly, we studied two SNPs in ~1200 samples; thus, the statistical power is markedly lower than in reported GWAS. Admittedly, most of the observed nominal significant associations between SNPs and traits related to adverse FD or obesity would not withstand corrections for multiple testing.

## 4. Materials and Methods

### 4.1. Subjects

The cohort includes a total of 2860 metabolically well-characterized participants (Table 2) of the Leipzig Obesity BioBank, recruited at four bariatric surgery centers in Leipzig, Karlsruhe, Dresden, and Gera (all in Germany). All subjects underwent routine clinical phenotyping as described previously [34,35]. All subjects had a stable weight, defined as fluctuations of <2% of body weight for at least 3 months before surgery. According to ADA criteria [36], 1034 subjects were diagnosed with type 2 diabetes (T2D), and 1826 had normal glucose tolerance (NGT). Furthermore, patients who had acute or chronic hepatic, inflammatory, infectious, or neoplastic diseases were excluded from the study. The study was approved by the ethics committee of the University of Leipzig (approval numbers: 159-12-21052012, 017-12-23012012). The study design follows the Declaration of Helsinki, and all participants gave written informed consent prior to participation.

### 4.2. COBLL1 and GRB14 mRNA Expression Analyses in AT

Paired samples of abdominal omental (visceral, vis) and sc AT were obtained from 560 Caucasian men (*n* = 149) and women (*n* = 411) who underwent open abdominal surgery as described previously [37]. The age ranged from 18 to 85 years and BMI from 18.5 to 90 kg/m^2^. After surgery, AT was immediately frozen in liquid nitrogen and stored at −80 °C. RNA was extracted from AT by using RNeasy Lipid Tissue Mini Kit (Qiagen, Hilden, Germany), and quantitative (q) PCR was performed as described [37,38]. Real-time qPCR was performed with the TaqMan Assay predesigned by Applied Biosystems (Foster City, CA, USA) for the detection of human *GRB14* (Hs00938593_m1), *COBLL1* (Hs01117513_m1), and *glyceraldehyde 3-phosphate dehydrogenase* (*GAPDH*) (Hs 02786624_g1) mRNA expression in AT. All reactions were carried out in 96-well plates using the QuantStudioTM 6 Flex System Fast Real-Time PCR system. *COBLL1* and *GRB14* mRNA expression was calculated relative to *GAPDH* mRNA expression.

### 4.3. Genotyping

Genotyping of the two SNPs rs10195252 and rs6738627 was conducted according to the manufacturer’s protocol using the SNP genotyping probes C_10003489_10 (rs6738627) and C_121021_10 (rs10195252) (Thermo Fisher Scientific, Waltham, MA, USA) and the ABI PRISM 7500 Sequence Detecting System (Life Technologies, Foster City, CA, USA). To control genotyping quality, a random selection of about 5% of samples were re-genotyped for both SNPs. All genotypes matched the initial designated genotypes. Genotype distributions were in Hardy–Weinberg equilibrium, all presenting *p* > 0.05.

### 4.4. Mediation Analysis

To test for the causative relationship between the genetic variant (independent variable), the expression of either *GRB14* or *COBLL1* (mediator), and the dependent variable (outcome of correlation analysis), the simple mediation model of the SPSS macro by Preacher and Hayes was used (Model 4, including the Sobel Test for testing the significance of the direct and indirect effects; bootstrapping procedure of 5000 samples providing 95% confidence interval (CI)).

### 4.5. In Vitro Grb14 and Cobll1 Knockdown in Murine Epididymal and Inguinal Cell Lines

Adipose tissue of newborn FVB mice was extracted and immortalized using the SV40 T antigen as described in detail elsewhere [39]. These immortalized epididymal and inguinal adipocytes were cultured and differentiated according to the reported protocols [39,40]. Epididymal fat was considered as the visceral adipose depot and inguinal was considered as the subcutaneous fat depot. Shortly, cells were grown in Dulbecco’s modified Eagle’s medium (high glucose) supplemented 20% fetal bovine serum at 37 °C and 5% CO_2_ until reaching 80% confluence. Three days prior to induction, electroporation transfection of siRNA into the cell was performed using Neon^®^ Transfection System. Subsequently, induction was initiated by adding 0.125 mM indomethacine, 2 µg/mL dexamethasone, and 0.5 mM isobutylmethylxanthine to the growth medium for 24 h and for differentiation; growth medium was supplemented with 20 mM insulin and 1 nM triiodthyronine. A second chemical transfection method was perfomed one day after the induction. In this case, the transfection Reagent DharmaFECT^®^ was used. Cells were grown for 8 days in differentiation medium. Thus, cells were harvested at time point: 80% confluence (=day−2), day 0 (=day of induction), day 2, 4, 6, 8 (=2, 4, 6, 8 days after induction), and washed and frozen immediately at −80 °C until RNA or protein extraction. All differentiation lines were run in triplicate. Adipocyte differentiation and lipid droplet accumulation was monitored by AdipoRedTM staining (Lonza, Basel Switzerland); siRNA for *Grb14* (Horizon, Catalog ID: L-044828-00-0005), *Cobll1* (Horizon, Catalog ID: L-051032-01-0005), and a negative control (ON-TARGETplus Non-Targeting Pool, Horizon, Catalog ID: D-001810-10-20) were used. *Grb14* and *Cobll1* knock-downs were validated at mRNA and protein level. The mRNA knockdown efficiency was 40% and 50% knock-down in inguinal and epididymal, respectively (Figure S1).

### 4.6. Statistical Analysis

Statistical tests were performed using the IBM SPSS Statistic 25 software (IBM Corp., Armok, NY, USA). Normal distribution of variables included in this study was checked prior statistical analyses and logarithmically transformed to achieve approximate normal distribution. Differences in mRNA expression between vis and sc AT were assessed using the paired Student’s *t*-test. Unpaired t-Test was used to analyze differences in adipose tissue mRNA expression among the groups. Logistic regression analyses have been conducted for the association of the SNP and the obesity/diabetes status. Linear regression analyses were used to assess the relationship between genetic variants/mRNA expression levels and quantitative metabolic traits. Pearson’s correlation analyses were conducted using two-way bivariate correlations. The additive model (with genotypes coded to 0, 1, and 2) was used, and if not stated otherwise, all *p*-values are adjusted for age, sex, and BMI. Two-sided *p*-values ≤ 0.05 were considered to provide evidence for nominal association and are presented without correction for multiple hypothesis testing.

## 5. Conclusions

Our data support the role of *GRB14/COBLL1* gene expression in body fat distribution (vis/sc) and the *GRB14/COBLL1* locus in metabolic sequelae of adverse fat distribution. In particular, their role in lipid metabolism and glucose homeostasis is likely to be mediated by *GRB14* gene expression in visceral AT. Considering the mediation, which is largely based on statistical modeling with genetic variants, it will now be inevitable to confirm these findings in functional experiments in vitro and/or in vivo to better understand molecular mechanisms underlying the SNP associations with adverse body FD.

## Figures and Tables

**Table 1:** Case control study for type 2 diabetes.

Genetic Variants	NGT	T2D	MAF NGT/T2D	*p*-Value (Add Model) adj. for Age, Sex, BMI (OR [95% CI])	(a) *p*-Value (Add Model) adj. for Age, Sex, BMI (OR [95% CI])
rs6738627 (G/A)					
GG	215 (36.7%)	176 (38.1%)	0.4/0.36	0.018(1.269 [1.041–1.547])	0.005 (1.85 [1.21–2.83])
AG	270 (46.1%)	235 (50.9%)			0.006 (1.78 [1.18–2.68])
AA	101 (17.2%)	51 (11%)			
rs10195252 (T/C)					
CC	132 (19%)	94 (17%)	0.43/0.42	0.339 (1.09 [0.914–1.3])	
TC	334 (47.8%)	285 (52%)			
TT	233 (33.2)	174 (31%)			

NGT: subjects with normal glucose tolerance; T2D: subjects with type 2 diabetes; MAF: minor allele frequency; OR: odds ratio for the minor allele; CI: confidence interval; add: additive model; adj: adjusted; (a): T2D logistic regression for rs6738627, AA as an indicator, adjusted for sex, age, and BMI.

**Table 2:** Anthropometric and metabolic characteristics of the studied cohort.

	BMI < 30 kg/m^2^	BMI 30~40 kg/m^2^	BMI ≥ 40 kg/m^2^
	(N = 256)	(N = 354)	(N = 2250)
Age (years)	61 ± 15	50 ± 13	46 ± 12
Women/Men (*n*)	134/122	248/106	1585/665
T2D (*n*)	36	118	880
Body weight (kg)	72 ± 11.6	104.4 ±14.7	146.7 ± 27.4
Height (m)	1.7 ± 0.1	1.7 ± 0.1	1.7 ± 0.1
BMI (kg/m^2^)	25.0 ± 2.8	36.2 ± 2.8	50.6 ± 7.8
Waist circumference (cm)	89.7 ± 17.9	116.6 ± 14.8	142.5 ± 17.4
Hip circumference (cm)	94.6 ± 12	118.3 ± 12.9	148.8 ± 17
WHR	0.9 ± 0.1	1 ± 0.1	1 ± 0.1
Body fat (%)	23.5 ± 5.3	38 ± 8.8	48.1 ± 8.4
FPG (mmol/L)	5.7 ± 1.2	6.2 ± 2.6	6.6 ± 2.7
FPI (pmol/L)	56.3 ± 74.8	109.3 ± 143	153 ± 136.5
HbA1c (%)	5.6 ± 0.6	5.9 ± 0.9	6.2 ± 1.3
HbA1c (mmol/mol)	29.4 ± 2.4	40.1 ± 11.8	43.3 ± 15.2
Total cholesterol (mmol/L)	5.2 ± 1	5.1 ± 1.2	4.9 ± 1.1
HDL-C (mmol/L)	1.4 ± 0.4	1.3 ± 0.4	1.2 ± 0.5
LDL-C (mmol/L)	3.1 ± 0.9	3.3 ± 1	3.1 ± 0.9
Triglycerides (mmol/L)	1.2 ± 0.6	1.7 ± 1.1	1.9 ± 1.4
AT *COBLL1* mRNA expression *n* (%)	19 (3)	38 (7)	467 (90)
AT *GRB14* mRNA expression *n* (%)	18 (3)	44 (8)	498 (89)
rs10195252 (T > C) carrier *n* (%)	131 (10)	146 (11)	1106 (79)
rs6738627 (G > A) carrier *n* (%)	133 (13)	123 (12)	870 (75)

Data are given as means ± SD. AT: adipose tissue; BMI: body max index; WHR: waist-to-hip ratio; FPG: fasting plasma glucose; FPI: fasting plasma insulin; HbA1c: glycated haemoglobin; HDL-C: high-density lipoprotein cholesterol; LDL-C: low density lipoprotein cholesterol; *n*: number.

## Data Availability

Not applicable.

## Supplementary Materials

The following supporting information can be downloaded at: https://www.mdpi.com/article/10.3390/ijms23158558/s1.
