# Supplementary material for: Metabolic Effects of the Waist-To-Hip Ratio Associated Locus GRB14/COBLL1 Are Related to GRB14 Expression in Adipose Tissue
Source: Int J Mol Sci. 2022 Aug 2;23(15):8558. doi: 10.3390/ijms23158558 (PMC9369072; doi:10.3390/ijms23158558)
Supplement: Supplementary file 1 [file ijms-23-08558-s001.zip › ijms-1832152-SI.pdf]

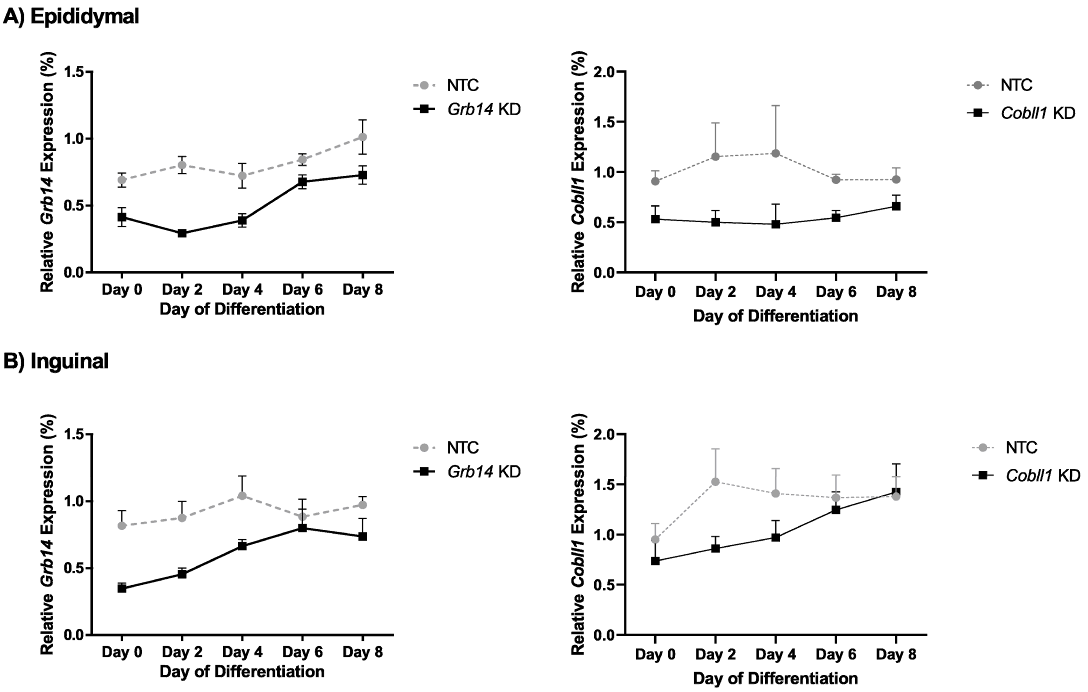

Figure S1. Relative mRNA expression of *Grb14* and *Cobll1* in mouse cells. A) in epididymal cells; B) in inguinal cells. Relative mRNA expression normalized to blank comparison group. NTC: non-silencing siRNA group. KD: knock-down.
